# Limit of detection in different matrices of 19 commercially available rapid antigen tests for the detection of SARS-CoV-2

**DOI:** 10.1038/s41598-021-97489-9

**Published:** 2021-09-15

**Authors:** Ana I. Cubas-Atienzar, Konstantina Kontogianni, Thomas Edwards, Dominic Wooding, Kate Buist, Caitlin R. Thompson, Christopher T. Williams, Edward I. Patterson, Grant L. Hughes, Lisa Baldwin, Camille Escadafal, Jilian A. Sacks, Emily R. Adams

**Affiliations:** 1 Liverpool School of Tropical Medicine, Centre for Drugs and Diagnostics, Liverpool, L3 5QA UK; 2 Departments of Vector Biology and Tropical Disease Biology, Liverpool School of Tropical Medicine, Centre for Neglected Tropical Diseases, Liverpool, L3 5QA UK; 3 Department of Biological Sciences, Brock University, St. Catharines, L2S 3A1 Canada; 4 FIND, Foundation for Innovative New Diagnostics, Geneva, Switzerland

**Keywords:** Viral infection, Immunochemistry, Laboratory techniques and procedures

## Abstract

In the context of the coronavirus disease 2019 (COVID-19) pandemic there has been an increase of the use of antigen-detection rapid diagnostic tests (Ag-RDT). The performance of Ag-RDT vary greatly between manufacturers and evaluating their analytical limit of detection (LOD) has become high priority. Here we describe a manufacturer-independent evaluation of the LOD of 19 marketed Ag-RDT using live SARS-CoV-2 spiked in different matrices: direct culture supernatant, a dry swab, and a swab in Amies. Additionally, the LOD using dry swab was investigated after 7 days’ storage at − 80 °C of the SARS-CoV-2 serial dilutions. An LOD of ≈ 5.0 × 10^2^ pfu/ml (1.0 × 10^6^ genome copies/ml) in culture media is defined as acceptable by the World Health Organization. Fourteen of 19 Ag-RDTs (ActiveXpress, Espline, Excalibur, Innova, Joysbio, Mologic, NowCheck, Orient, PanBio, RespiStrip, Roche, Standard-F, Standard-Q and Sure-Status) exceeded this performance criteria using direct culture supernatant applied to the Ag-RDT. Six Ag-RDT were not compatible with Amies media and a decreased sensitivity of 2 to 20-fold was observed for eleven tests on the stored dilutions at − 80 °C for 7 days. Here, we provide analytical sensitivity data to guide appropriate test and sample type selection for use and for future Ag-RDT evaluations.

## Introduction

During the Severe Acute Respiratory Syndrome Coronavirus 2 (SARS-CoV-2) pandemic, reverse transcription polymerase chain reaction (RT-PCR) has become the gold standard for diagnosis of acute infection^1^. However, RT-PCR technologies have several limitations: they are not deployed easily, require significant laboratory infrastructure, reagents, and skilled staff, and during this pandemic shortages in global supply have presented challenges^2–4^. In addition, the turnaround time from sample collection to result can be up to 72 h compromising the effectiveness of triage, isolation, and contact tracing strategies^5,6^. In comparison, rapid diagnostic tests (RDTs) based on antigen detection (Ag-RDT) can determine the presence of the virus in a clinical sample on site in less than 30 min without the need for a laboratory. Ag-RDTs are faster, cheaper and can be available at the point-of-care (POC), which is especially important for implementation in community and low-resource settings, where limited laboratories and trained staff are available, and there may be suboptimal cold chain capacity to ensure appropriate conditions for more complex testing^7–9^. Furthermore, their use could also enable rapid isolation of cases and their contacts.

Ag-RDTs are less sensitive than RT-PCR, but clinical evaluation data is emerging that demonstrates Ag-RDTs are accurate at detecting the vast majority of individuals with a high-viral load (cycle threshold (Ct) on RT-PCR ≤ 25.0 or > 10^6^ genomic virus copies/ml)^7,10–16^. In addition, in outbreak scenarios, a diagnostic test with lower sensitivity but a fast result enables quick interventions such as self-isolation and isolating contacts of cases^17^. Implementation of Ag-RDTs into testing algorithms would allow rapid detection and isolation of new cases and thereby support the test, trace and isolate strategy, aiming to stop transmission chains and reduce the impact of COVID-19.

Ag-RDTs have been recently used for screening asymptomatic people in high prevalence areas and frontline workers to quickly identify persons with a SARS-CoV-2 infection to adapt infection prevention and control measures, thus preventing transmission in the community^18–20^. A mass testing program screening asymptomatic people in Slovakia using Ag-RDTs was shown to reduce the prevalence of SARS-CoV-2 infections by > 50% within 2 weeks^18^.

Despite the increased adoption of Ag-RDTs as an alternative of RT-PCR, independent analytical sensitivity data is currently lacking for many rapid antigen tests. Evaluation of Ag-RDTs using spiked samples in the laboratory before proceeding on clinical specimens is of paramount importance because the sensitivity of Ag-RDTs is highly variable depending on the manufacturer, ranging 0–95% in respiratory samples^7,21–24^.

Here we describe a single-center, manufacturer-independent analytical validation of 19 commercially available Ag-RDTs. The aims of the study were to assess the limit of detection (LOD) using viral culture in different sample matrices: direct culture supernatant, dry swab and swabs in Amies. The effect on the LOD of one freeze–thaw cycle following storage at − 80 °C was also explored; demonstration of adequate performance using this sample type could support future rapid evaluation of Ag-RDTs with stored material.

## Results

### LOD using different matrices

The LOD was evaluated in three matrices: direct culture supernatant, dry swab and swab in Amies. Direct viral culture supernatant was used as it is the standardized protocol for the evaluation of LOD in Ag-RDTs^25^. Dry swab matrix using the proprietary swab kit was selected to evaluate the LOD in the sample type as defined in the instructions for use (IFU). Finally, swab in Amies was used to assess the use of the same swab used for RT-PCR as a sample type for Ag-RDTs.

**Figure 1:**
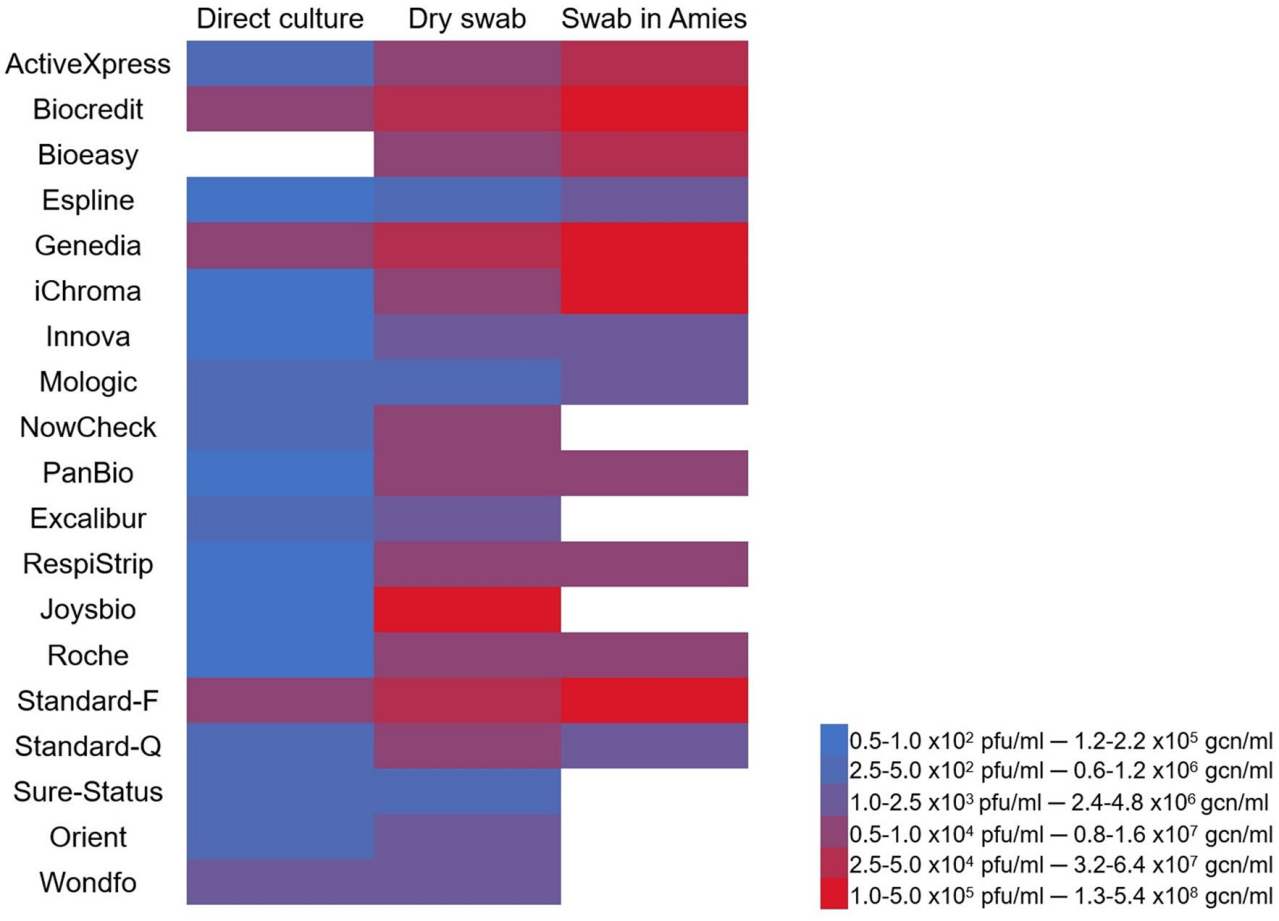
Heatmap of the LODs on all matrices. Ag-RDTs in direct culture matrix colored blue fulfilled WHO criteria. Note: no colored cells for direct culture indicates no Ag-RDT was tested with that matrix, no colored cells in swab in Amies swab means interference with that matrix, hence LOD is not available.

A predefined performance criterion of an analytical LOD of ≤ 5.0 × 10^2^ plaque forming units (pfu)/ml (≈ 10^6^ genome copy numbers (gcn)/ml) using direct culture supernatant was selected based on current WHO and national standards^25,26^. Fourteen of 19 Ag-RDTs evaluated in this study had an LOD of ≤ 5.0 × 10^2^ pfu/ml (ActiveXpress, Espline, Excalibur, Innova, Joysbio, Mologic, NowCheck, Orient, PanBio, RespiStrip, Roche, Standard-F, Standard-Q and Sure-Status) using direct culture supernatant and the remaining had an LOD of 1.0–5.0 × 10^3^ pfu/ml (Biocredit, Genedia, iChroma and Wondfo) (Fig. 1). Espline, iChroma, Innova Panbio and Roche had the lowest LOD using direct culture supernatant reaching 0.5–1.0 × 10^2^ pfu/ml, followed by ActiveXpress, Excalibur, Mologic, Orient, Standard-Q, Sure-Status and Respi-Strip, with an LOD of 2.5–5.0 × 10^2^ pfu/ml. Biocredit, Genedia, Standard-F and Wondfo and were the least sensitive with an LOD of 1.0–5.0 × 10^3^ pfu/ml. See Fig. 1 and Supplementary Table S1 in Supplementary Information for detailed LODs on all Ag-RDTs.

The LOD using dry swab was poorer in all tests when compared to direct culture supernatant with the exception of Mologic, where the LOD was the same using direct culture supernatant and dry swab. The LOD using dry swab was ≤ 5.0 × 10^2^ pfu/ml only in four Ag-RDT (Espline, Mologic, Roche and Sure-Status), 1.0–5.0 × 10^3^ pfu/ml in seven (Bioeasy, Innova, NowCheck, Orient, Panbio, Respi-Strip and Standard-Q) and ≥ 1.0 × 10^4^ pfu/ml in seven (ActiveXpress, Biocredit, Genedia, iChroma, Joysbio, Standard-F and Wondfo). The least sensitive using dry swab matrix was Joysbio with an LOD of 2.5 × 10^5^ pfu/ml (Fig. 1).

None of the Ag-RDTs evaluated specifically indicate compatibility with swabs in Amies media. However, four tests recommend the use of universal or viral transport media (UTM/VTM) (Biocredit, Respi-Strip, Roche and Wondfo), three tests do not recommend the use of UTM/VTM (NowCheck, Standard-F and Standard-Q), and the remaining kits do not mention the use of any transport media. Six Ag-RDTs (Excalibur, Joysbio, NowCheck, Orient, Sure-Status and Wondfo) were found to be incompatible with the Amies media, as these showed a positive test line with the negative control sample. Of these, Wondfo is the only kit which recommends the use of transport media. LODs using swabs in Amies media was poorer than using dry swabs except in two tests where the LOD was the same as with the dry swabs (PanBio and Standard-Q).

### Effect of swab absorbance and volume of extraction buffer in the LOD of dry swabs

We investigated whether the absorbance of the proprietary swabs provided with the Ag-RDT kits affected the LOD compared with direct culture supernatant i.e. if a less absorbent swab resulted in a poorer LOD in dry swab compared with the LOD obtained in direct culture supernatant for the same test. We also investigated the effect of the volume of the extraction buffer as this varied per Ag-RDT kit i.e. if larger volumes of extraction buffer resulted in poorer LODs due to the dilution factor. The volume recovered by swabs per tests and volumes of extraction buffer per kit are shown in Supplementary Table S2 in Supplementary Information. Spearman’s correlation coefficient did not show any statistically significant correlation between the LOD and the volume recovered by swabs (P = 0.421, ρ = − 0.50), volume of extraction buffer (P = 0.483, ρ = − 0.011) and a combination of both (P = 0.460, ρ = − 0.025).

### LOD one freeze–thaw cycle after 7 days at − 80 °C

Ag-RDTs are intended to be POC tests and thus the majority are recommended for use with freshly collected specimens. To validate test performance, use of stored material is much easier. Therefore, we performed this experiment to understand whether LOD is impacted following sample storage. The LOD of each of the tests using SARS-CoV-2 serial dilutions after 7 days storage at − 80 °C and one freeze–thaw cycle is shown in Table 1.

**Table 1:** LOD after 1 week − 80 °C storage and one freeze-thawed cycle.

Test	LOD dry swab	LOD after − 80 °C storage
ActiveXpress	1.0 × 10^4^	1.0 × 10^4^
Biocredit	5.0 × 10^4^	1.0 × 10^5^
Bioeasy	5.0 × 10^3^	5.0 × 10^3^
Espline	5.0 × 10^2^	1.0 × 10^3^
Genedia	2.5 × 10^4^	5.0 × 10^5^
IChroma	1.0 × 10^4^	1.0 × 10^4^
Innova	1.0 × 10^3^	1.0 × 10^4^
Mologic	2.5 × 10^2^	1.0 × 10^3^
NowCheck	5.0 × 10^3^	5.0 × 10^3^
PanBio	5.0 × 10^3^	2.5 × 10^3^
Excalibur	1.0 × 10^3^	5.0 × 10^3^
RespiStrip	5.0 × 10^3^	5.0 × 10^3^
Joysbio	2.5 × 10^5^	1.0 × 10^5^
Roche	5.0 × 10^2^	1.0 × 10^3^
Standard-F	2.5 × 10^4^	5.0 × 10^5^
Standard-Q	5.0 × 10^3^	1.0 × 10^3^
Sure-Status	5.0 × 10^2^	1.0 × 10^3^
Orient	2.5 × 10^3^	1.0 × 10^4^
Wondfo	2.5 × 10^3^	2.5 × 10^3^

The LOD for 6 tests (ActiveXpress, Bioeasy, iChroma, NowCheck, RespiStrip and Wondfo) was equivalent using stored, frozen viral dilutions compared to fresh preparations. Three tests showed increased sensitivity (lower LOD by two- to five-fold) (Joysbio, PanBio and Standard-Q), and eleven showed poorer performance with a higher LOD of twofold (Biocredit, Espline, Roche and Sure-Status), 4–5 (Excalibur, Mologic and Orient), ten (Innova) and twenty (Genedia and Standard-F).

## Discussion

Here, we present the analytical performance of 19 antigen rapid tests, which are currently on the market and in use in multiple countries. Analytical LODs are a useful proxy of clinical sensitivity, and the most standardized way to evaluate multiple antigen tests head-to-head, as each test requires a separate swab from patients. An approximate LOD of ≤ 5.0 × 10^2^ pfu/ml (≈ 1.0 × 10^6^ copies/ml) calculated using direct culture supernatant, has been proposed as the minimal analytical sensitivity by the WHO and the Department of Health and Social Care (DHSC, U.K.)^25,26^. Fourteen of the 19 marketed Ag-RDTs evaluated in this study fulfill this requirement (ActiveXpress, Bioeasy, Espline, Innova, Mologic, NowCheck, PanBio, Excalibur, RespiStrip, Joysbio, Roche, Standard-F, Standard-Q, Sure-Status and Orient).

Evaluation of the LOD using the kit-specific swabs immersed in the viral culture dilutions offers a more representative comparison to the level of sensitivity for clinical samples than using direct viral culture as these Ag-RDT kits are used by applying the kit-specific swabs to obtain a respiratory sample and the swab is immersed into the kit’s extraction buffer. Four of the 19 tests detected samples with concentrations ≤ 5.0 × 10^2^ pfu/ml (≈ 1.0 × 10^6^ gcn/ml) when using the dry swabs (Mologic, Espline, Roche, Sure-Status) and none of the tests met that LOD target when using swabs in Amies, likely due to the dilution factor with the addition of 1 ml Amies buffer as well as potential chemical interactions between the media and the kit-specific buffers.

It is expected that the more absorbent swabs will absorb more viral material and the lower volume of extraction buffer will result in more concentrated sample within the test, and so we investigated if the volume recovered by the swab and the volume of extraction buffer had any bearing on the LOD of each test when compared with the LOD achieved with direct culture supernatant. We took into account the volume recovered from the swab and the volume of proprietary buffer provided, but no correlation was found, this suggests that other factors may reduce the sensitivity when using swabs such as differences in the formulation of the proprietary buffers and efficiency of sample recovery from the swab (in natural conditions). A reduction in sensitivity using clinical samples may be observed compared to swabs in viral culture, as clinical samples are more viscous than culture media, potentially resulting in less viral material being absorbed onto the swab. The efficiency of the recovery is also likely to be increased by the centrifugation method used in our protocol.

We also evaluated the LOD and compatibility of the Ag-RDTs using a swab placed in Amies media, as these are routinely used to collect upper-respiratory samples in SARS-CoV-2 suspected individuals for diagnosis using RT-PCR^27,28^. If the same swab proves to be suitable for both RT-PCR and rapid antigen testing, one swab can be used for both tests as part of a serial algorithm. As well, the frozen leftover Amies media/swab from RT-PCR testing could be used for future Ag-RDT evaluations. Either approach could simplify clinical and/or evaluation workflows. None of the Ag-RDT manufacturers specifically recommend the use of Amies media, and we demonstrated here that this ‘off-label’ sample preparation should be used with caution: six tests had false positive results (Espline, Excalibur, Joysbio, Sure-Status and Orient) and sensitivity was also reduced due to the additional volume of Amies.

The effect of storage at − 80 °C and one freeze–thaw cycle was evaluated, with eleven Ag-RDTs showing a loss of sensitivity by up to 20-fold. A small decrease in sensitivity has been reported in in SARS-CoV-2 RT-PCR testing (< 1 RT-PCR cycle threshold) after one and two freeze–thaw cycles^29^ but there are no studies so far that have reported the effect of freeze–thaw on antigen detection. Results here highlight that the use of frozen material with Ag-RDTs should be performed with caution. The fact that three tests showed two-to-five-fold better sensitivity after an additional freeze thaw-cycle could not be explained in here, a further investigation is required with a larger sample size to rule out whether this phenomenon was within the margin of error of the experiment.

Three out of 19 Ag-RDTs (Bioeasy, iChroma and Standard-F) rely on detection of a fluorescent signal using a reader. Though this may enable quantitative detection and potentially more consistent result interpretation, we did not find any improved sensitivity for this test format. Furthermore, Ag-RDTs that rely on a device may limit testing throughput if only one test can be read at a time. The reader also presents additional costs, as well as potential technical and maintenance issues which can be a barrier to implementation.

This analytical study has some limitations, as only a single isolate (REMRQ0001/Human/2020/Liverpool) was used to assess the LODs but our results are consistent with other recently-published analyses^30,31^. To the authors knowledge, all 19 tests evaluated here detect the nucleoprotein, presumably chosen for abundance and relative low mutation rate and therefore hypothesized to pick up all currently known variants^32,33^. Another limitation is that only one lot per kit was evaluated.

There is a growing number of studies suggesting that although antigen detection is less analytically sensitive than nucleic acid amplification techniques, it may strongly correlate with culturable virus, which may be a proxy for transmissibility. Hence Ag-RDTs could be informative for test, trace, isolate processes for the most infectious individuals^10–15^. Viral loads have been estimated to range from 10^8^ to 10^11^ gcn/ml in the most infectious patients^34–36^. The majority of Ag-RDTs evaluated here have an LOD predicted to successfully diagnose infected individuals with higher viral loads in this range across all matrices, except Joysbio that had an LOD of 5.4 × 10^8^ gcn/ml in dry swab. Further, Biocredit, iChroma, Standard-F and Genedia tests had LOD greater than 1.0 × 10^8^ gcn/ml when using swabs placed in Amies.

In conclusion, the most sensitive tests with an LOD ≤ 5.0 × 10^2^ pfu/ml (≈ 1.2 × 10^6^ gcn/ml) on dry swabs and direct culture supernatant were Espline, Mologic, Sure-Status and Roche and the least sensitive on all matrices were Biocredit, iChroma, Standard-F and Genedia. The differences of LODs found here between tests and/or matrices ranged between 2–3 logs (i.e. 100–1000 fold). Some tests showed impaired performance when using freeze–thaw material and/or Amies media. These findings highlight the importance of understanding assay specific performances and the need to select the appropriate sample matrix and the right test for each intended use, particularly for laboratories and evaluation programs that seek a rapid validation of Ag-RDT using frozen stored samples and ‘off-label’ specimen preparations. The LODs obtained in this comprehensive assessment of analytical sensitivity are consistent with rapidly emerging clinical performance data^9,11,37^ demonstrating the high clinical accuracy of Ag-RDTs for rapid detection of individuals with high viral loads, which can be very impactful for initiation of isolation and tracing measures.

## Methods

### Ethical statement

As the study involves only the data based on SARS-CoV-2 spiked samples and no clinical and tissue samples are used, ethical approval is not required for this study.

### Evaluated Ag-RDTs

Nineteen Ag-RDT based on lateral flow principle were evaluated in this study (Table 2): (1) ActivXpress + COVID-19 Ag Complete Kit (Edinburgh Genetics Ltd), referred to as ActivXpress+, (2) Biocredit COVID-19 Ag (Rapidgen Inc.), referred to as Biocredit, (3) Bioeasy 2019-nCoV Ag (Shenzhen Bioeasy Biotechnology), referred to as Bioeasy, (4) Espline SARS-CoV-2 (Fujirebio Diagnostics Inc.), referred to as Espline, (5) Genedia W COVID-19 Ag (Green Cross Medical Science), referred to as Genedia, (6) iChroma COVID-19 Ag Test (Boditech Medical Inc.), referred to as iChroma, (7) Innova SARS-CoV-2 Antigen Rapid (Innova Medical Group Ltd.), referred to as Innova, (8) Mologic COVID-19 Ag Test device (Mologic Ltd), referred to as Mologic, (9) NowCheck COVID-19 Ag test (Bionote Inc.), referred to as NowCheck, (10) Panbio COVID-19 Ag Rapid Test (Abbott Rapid Diagnostics), referred to as Panbio, (11) Rapid SARS-CoV-2 Antigen test card (Excalibur Healthcare Services), referred to as Excalibur, (12) Respi-Strip COVID-19 Ag (Coris Bioconcept), referred to as Respi-Strip, (13) SARS-CoV-2 Antigen Rapid Test Kit (Joysbio Biotechnology Ltd.), referred to as Joysbio, (14) SARS-CoV-2 Rapid Antigen Test (co-developed by SD Biosensor Inc and Roche Diagnostics, distributed by Roche Diagnostics), referred to as Roche, (15) Standard-F COVID-19 Ag FIA (SD Biosensor Inc), referred to as Standard-F, (16) Standard-Q COVID-19 (SD Biosensor Inc), referred to as Standard-Q, (17) Sure Status COVID-19 Antigen Card Test (Premier Medical Corporation), referred to as Sure-Status, (18) Coronavirus Ag Rapid Test (Zhejiang Orient Gene Biotech Ltd.), referred to as Orient, (19) Wondfo 2019-nCoV Antigen Test (Guangzhou Wondfo Biotech Co.), referred to as Wondfo. The selection of the Ag-RDT was based on expression of interest via the Infection Innovation Consortium (iiCON) and Foundation of New Diagnostics (FIND). Companies had no involvement in the design or reporting of the study.

**Table 2:** Characteristics of the Ag-RDT tested.

Test name in this study	Assay	Manufacturer/Distributor	Country	Recommended sample	Principle	Format	Minutes to result
ActiveXpress	ActivXpress + COVID-19 Ag Complete Kit	Edinburgh Genetics Ltd	UK	NP/OP swab	Colloidal gold	Cassette	15–20
Biocredit	Biocredit COVID-19 Ag	Rapidgen Inc	Rep. Korea	NP swab/UTM/VTM	Colloidal gold	Cassette	30
Bioeasy	Bioeasy 2019-nCoV Ag	Shenzhen Bioeasy Biotechnology	China	NP swab	Fluorescence	Cassette	10
Espline	ESPLINE SARS-CoV-2	Fujirebio Diagnostics Inc	Japan	NP swab	Colloidal gold	Cassette	30
Excalibur	Rapid SARS-CoV-2 Antigen test card	Boson Diagnostics/Excalibur Healthcare Services	UK	NP swab	Colloidal gold	Cassette	15
Genedia	GENEDIA W COVID-19 Ag	Green Cross Medical Science	Rep. Korea	NP swab/Sputum	Colloidal gold	Cassette	5–10
iChroma	iChroma COVID-19 Ag Test	Boditech Medical Inc	Rep. Korea	NP swab	Fluorescence	Cassette	12
Innova	Innova SARS-CoV-2 Antigen Rapid	Innova Medical Group Ltd	UK	N/T swab	Colloidal gold	Cassette	15
Joysbio	SARS-CoV-2 Antigen Rapid Test Kit	Joysbio Biotechnology Ltd	China	N swab	Colloidal gold	Cassette	15–20
Mologic	Mologic COVID-19 Ag Test device	Mologic Ltd	UK	T/N/NT swab	Colloidal gold	Cassette	10
NowCheck	NowCheck COVID-19 Ag test	Bionote Inc./Mologic Ltd	Rep. Korea	NP swab	Colloidal gold	Cassette	15–30
Orient	Coronavirus Ag Rapid Test	Zhejiang Orient Gene Biotech Ltd	China	NP swab	Colloidal gold	Cassette	10–15
PanBio	Panbio COVID-19 Ag Rapid Test	Abbott Rapid Diagnostics	Rep. Korea	NP swab	Colloidal gold	Cassette	15
RespiStrip	Respi-Strip COVID-19 Ag	Coris Bioconcept	Belgium	NP wash/ UTM/VTM	Colloidal gold	Dipstick	15–30
Roche	SARS-CoV-2 Rapid Antigen Test	SD Biosensor Inc./ Roche Diagnostics)	Switzerland	NP swab/VTM/UTM	Colloidal gold	Cassette	15–30
Standard-F	Standard F COVID-19 Ag FIA	SD Biosensor Inc	Rep. Korea	NP swab	Fluorescence	Cassette	15–30
Standard-Q	Standard Q COVID-19	SD Biosensor Inc	Rep. Korea	NP swab	Colloidal gold	Cassette	15
Sure-Status	Sure-Status COVID-19 Antigen Card Test	Premier Medical Corporation	India	NP swab	Colloidal gold	Cassette	15–20
Wondfo	Wondfo 2019-nCoV Antigen Test	Guangzhou Wondfo Biotech Co	China	NP/OP swab/VTM	Colloidal gold	Cassette	10–15

*Rep.* Republic, *OP* oropharyngeal, *NP* nasopharyngeal, *N* nasal, *T* throat, *NT* nasal-throat, *UTM* universal transport media, *VTM* viral transport media.

### SARS-CoV-2 serial dilutions and quantification of copy numbers

The SARS-CoV-2 isolate REMRQ0001/Human/2020/Liverpool was propagated in Vero E6 cells (C1008; African green monkey kidney cells), maintained in DMEM with 2% fetal bovine serum (FBS) and 0.05 mg/ml gentamycin. Ten-fold serial dilutions of SARS-CoV-2 stock were made starting from 1.0 × 10^6^ pfu/ml to 1.0 × 10^2^ pfu/ml using culture media as a diluent (DMEM with 2% FBS % and 0.05 mg/ml gentamycin). Two-fold dilutions were made below the ten-fold LOD dilution to refine the LOD. For quantification, viral RNA was extracted using QIAmp Viral RNA mini kit (Qiagen, Germany) according to the manufacturer's instructions. The genome copies/ml (gcn/ml) were calculated using the COVID-19 Genesig RT-qPCR kit (PrimerDesign, UK). RT-qPCR testing was carried out using the Rotor-Gene Q (Qiagen, Germany), with a ten-fold serial dilution of using quantified specific in vitro-transcribed RNA^38^. A total of five replicates were tested for each standard curve point and extracted RNA from each culture dilution was tested in triplicate, and the gcn/ml was calculated from the mean Ct value of these replicates.

### Preparation of SARS-CoV-2 sample matrices and LOD testing protocol

Three types of sample matrices were tested (1) direct viral culture supernatant, (2) spiked dry swabs and (3) spiked wet swabs in Amies media.

For the direct viral culture matrix, a specific volume of the serial dilutions was added directly to the extraction buffers at a 1:10 ratio except for Respi-Strip which was added at 1:1 ratio with the extraction buffer following the IFU.

For dry swab testing, the proprietary nasopharyngeal (NP) or nasal (N) swabs included in each individual kit was used except for Respi-Strip, which does not include swabs, and the recommended Eswab (Copan, Italy) was used instead. To prepare the dry swab matrix, the swab was soaked in 1 ml of the virus culture dilution series for 6–8 s, followed by immersion in the prescribed amount of proprietary reaction buffer solution.

For the preparation of spiked wet swabs, Eswab in Amies media (Copan, Italy) was used across all tests. The swab was first immersed in the serial viral dilutions for 6–8 s, then placed into the Amies media to mimic the sample collection stage. Ag-RDTs were evaluated by then immersing the same swab into the extraction buffer, except for test Respi-Strip where 100 µl of the Amies was mixed at 1:1 with the extraction buffer following its IFU.

For all Ag-RDTs and matrices, the sample volumes applied, and procedures were performed as specified in the test specific IFUs.

The LOD was defined as the lowest dilution at which all three replicates were positive. Every dilution was tested in triplicate and non-spiked culture media and Amies were used as negative controls. Results were interpreted by two operators, each blinded to the result of the other. If a discrepant result was obtained, a third operator read any discrepant tests for a 2/3 result.

### Effect of swab absorbance and volume of extraction buffer in the LOD of dry swabs

We investigated the effect of the absorbance of the proprietary swabs and extraction buffer provided with the Ag-RDT kit in the LOD using dry swab, i.e. if a less absorbent swab and larger volumes of extraction buffer resulted in a poorer LOD on dry swab compared with direct culture supernatant. To compare the effectiveness of each NP and N swab to recover sample, the amount of liquid absorbed by the swabs was measured. Five replicates of each swab brand were immersed in culture media for 6–8 s, then taped on the inside of a 50 ml centrifuge tube. These were then centrifuged for 5 min at 1000*g* and the amount of liquid released was measured using a micropipette.

The degree of correlation of the difference between LOD of dry swabs and direct culture for the same Ag-RDT with the volume recovered by swab type and volume of proprietary were investigated by Spearman’s correlation coefficient rho (ρ). Statistical significance was set at P < 0.05.

### LOD after 7 days at – 80 °C and one freeze–thaw cycle

After performing the LOD experiments, the viral culture dilutions were stored at − 80 °C for 7 days and then the LOD experiments were performed again using the dry NP and N swabs. This would help to assess the use of stored clinical samples could be used to facilitate evaluation of Ag-RDTs.

## Supplementary Information

Supplementary Tables.

## Data Availability

All data generated during this study is presented in an analysed format in this manuscript. Raw datasets are available from the corresponding author on reasonable request.
