# Supplementary material for: Limit of detection in different matrices of 19 commercially available rapid antigen tests for the detection of SARS-CoV-2
Source: Sci Rep. 2021 Sep 15;11:18313. doi: 10.1038/s41598-021-97489-9 (PMC8443584; doi:10.1038/s41598-021-97489-9)
Supplement: Supplementary file 1 — Supplementary Tables. [file 41598_2021_97489_MOESM1_ESM.docx]

**Supplementary materials**

**Table S1. LOD obtained on different matrices.**

| **Test** | **Pfu/ml** | | | **Gcn/ml** | | |
| --- | --- | --- | --- | --- | --- | --- |
|  | Viral culture | Dry swab | Amies | Viral culture | Dry swab | Amies |
| ActiveXpress | 2.5 x10^2^ | 1.0 x10^4^ | 2.5 x10^4^ | 5.9 x10^5^ | 1.9 x10^7^ | 5.4 x10^7^ |
| Biocredit | 5.0 x10^3^ | 5.0 x10^4^ | 1.0 x10^5^ | 9.8 x10^6^ | 6.4 x10^7^ | 1.3 x10^8^ |
| Bioeasy |  | 5.0 x10^3^ | 2.5 x10^4^ |  | 8.1 x10^6^ | 3.2 x10^7^ |
| Espline | 5.0 x10^1^ | 5.0 x10^2^ | 2.5 x10^3^ | 1.1 x10^5^ | 1.2 x10^6^ | 4.9 x10^6^ |
| Genedia | 5.0 x10^3^ | 2.5 x10^4^ | 2.5 x10^5^ | 9.8 x10^6^ | 5.4 x10^7^ | 2.7 x10^8^ |
| iChroma | 1.0 x10^2^ | 1.0 x10^4^ | 5.0 x10^5^ | 2.2 x10^5^ | 1.6 x10^7^ | 5.4 x10^8^ |
| Innova | 1.0 x10^2^ | 1.0 x10^3^ | 1.0 x10^4^ | 2.2 x10^5^ | 2.4 x10^6^ | 1.9 x10^7^ |
| Mologic | 2.5 x10^2^ | 2.5 x10^2^ | 1.0 x10^4^ | 5.9 x10^5^ | 5.9 x10^5^ | 1.9 x10^7^ |
| NowCheck | 2.5 x10^2^ | 5.0 x10^3^ | Interference | 5.9 x10^5^ | 8.1 x10^6^ | ─ |
| PanBio | 1.0 x10^2^ | 5.0 x10^3^ | 5.0 x10^3^ | 2.2 x10^5^ | 8.1 x10^6^ | 9.8 x10^6^ |
| Excalibur | 2.5 x10^2^ | 1.0 x10^3^ | Interference | 5.9 x10^5^ | 2.4 x10^6^ | ─ |
| RespiStrip | 5.0 x10^2^ | 5.0 x10^3^ | 1.0 x10^4^ | 1.2 x10^6^ | 8.1 x10^6^ | 1.9 x10^7^ |
| Joysbio | 1.0 x10^2^ | 2.5 x10^5^ | Interference | 2.2 x10^5^ | 2.7 x10^8^ | ─ |
| Roche | 5.0 x10^1^ | 5.0 x10^2^ | 1.0 x10^4^ | 1.1 x10^5^ | 1.2 x10^6^ | 1.9 x10^7^ |
| Standard-F | 1.0 x10^3^ | 2.5 x10^4^ | 2.5 x10^5^ | 2.4 x10^6^ | 3.4 x10^7^ | 2.7 x10^8^ |
| Standard-Q | 2.5 x10^2^ | 5.0 x10^3^ | 5.0 x10^3^ | 5.9 x10^5^ | 8.1 x10^6^ | 9.8 x10^6^ |
| Sure-Status | 2.5 x10^2^ | 5.0 x10^2^ | Interference | 5.9 x10^5^ | 1.2 x10^6^ | ─ |
| Orient | 2.5 x10^2^ | 2.5 x10^3^ | Interference | 5.9 x10^5^ | 4.8 x10^6^ | ─ |
| Wondfo | 1.0 x10^3^ | 2.5 x10^3^ | Interference | 2.4 x10^6^ | 4.8 x10^6^ | ─ |

Abbreviations: gcn: genome copy numbers, pfu: plaque forming units

**Table S2. Details of volume recovered by swab, volume of extraction buffer per kit and LOD fold change comparing dry swab and direct culture.**

| **Test** | **Swab brand*** | **Volume recovered (µl)** | **Extraction buffer (µl)** | **Fold change LOD**  **dry swab-direct culture** |
| --- | --- | --- | --- | --- |
| ActiveXpress | Specimen Collection Swab | 49.4 | 260 | 40 |
| Biocredit | FLOQ SWABS COPAN (GREEN) | 62.8 | 400 | 10 |
| Bioeasy | MRC Miraclean Technology Co |  | 500 | 20 |
| Espline | PMWCORP | 53.6 | 200 | 10 |
| Genedia | NOBLE BIO | 29 | 340 | 5 |
| IChroma | PMWCORP | 53.6 | 100 | 100 |
| Innova | Disposable Sampling Swab | 50.1 | 350 | 10 |
| Mologic | iCLEAN (Nasal/Throat) | 118.6 | 300 | 1 |
| NowCheck | NOBLE BIO | 29 | 400 | 20 |
| PanBio | NOBLE BIO | 29 | 300 | 50 |
| Excalibur | Specimen Collection Swab | 49.4 | 250 | 4 |
| RespiStrip | Eswab | 126.2 | 100 | 10 |
| Joysbio | PMWCORP | 53.6 | 350 | 2500 |
| Roche | NOBLE BIO | 29 | 340 | 10 |
| Standard-F | iCLEAN (Nasopharyngeal) | 57.3 | 340 | 25 |
| Standard-Q | iCLEAN (Nasopharyngeal) | 57.3 | 340 | 20 |
| Sure-Status | NP Nylon Flocked swab | 28.2 | 300 | 2 |
| Orient | Disposable swab Jiangsu | 30.2 | 300 | 10 |
| Wondfo | FLOQ SWABS COPAN (BLUE) | 67 | 400 | 2.5 |

***** The experiments were performed once by swab type and no repeated in the event of a test using the same swab type.
